# Supplementary material for: Optimized Extraction of Bioactive Polysaccharides from Wild Mushrooms: Toward Enhanced Yield and Antioxidant Activity
Source: Molecules. 2025 Dec 3;30(23):4647. doi: 10.3390/molecules30234647 (PMC12693275; doi:10.3390/molecules30234647)
Supplement: Supplementary file 1 [file molecules-30-04647-s001.zip › molecules-4006494-supplementary.pdf]

Optimized Extraction of Bioactive Polysaccharides from Wild Mushrooms: Toward Enhanced Yield and Functionality

Aya Samy Ewesys Khalil, Marcin Lukasiewicz

Supplementary materials

1. Response Surface Plots

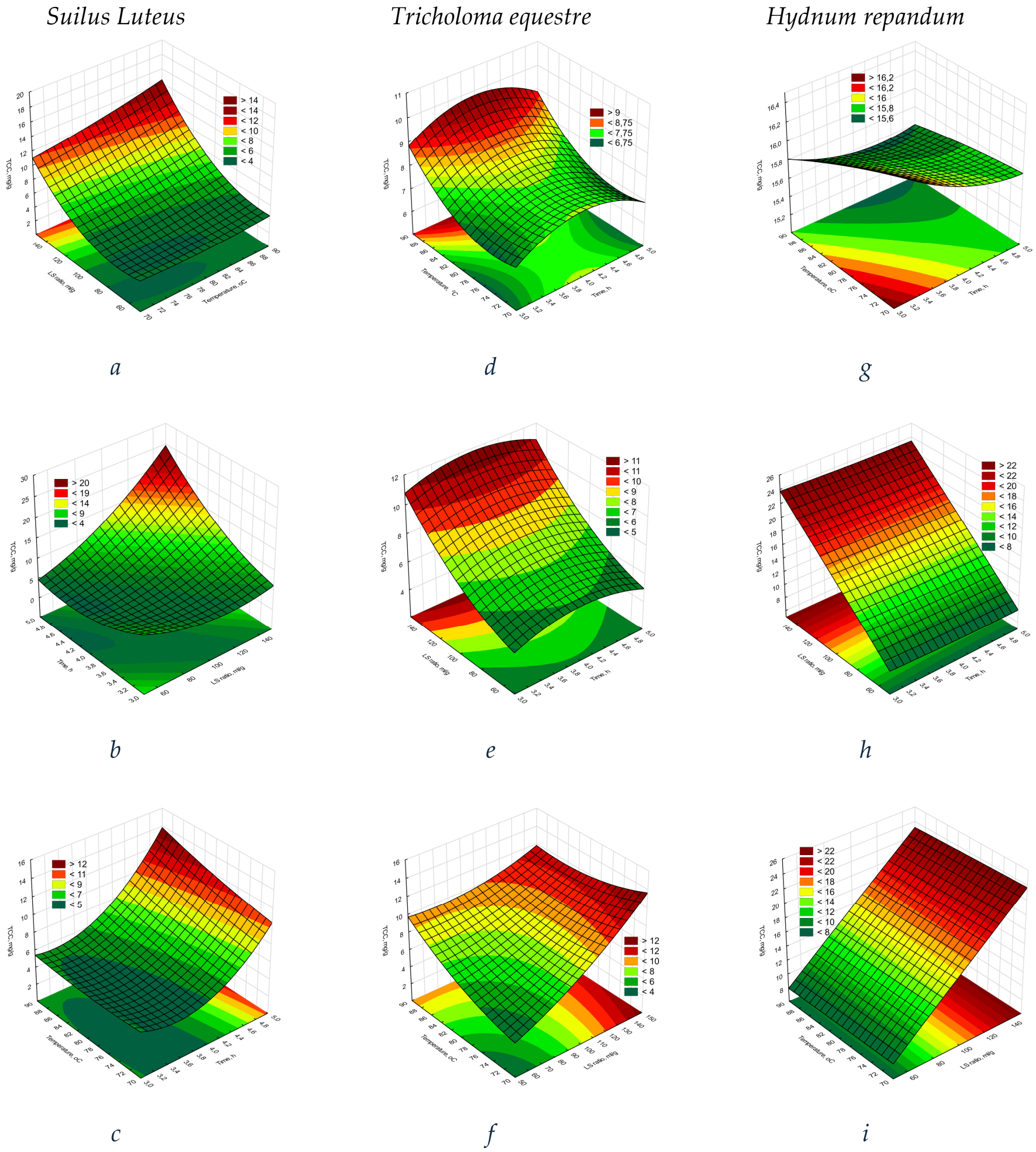

Figure 1S. Response surface plots for TCC

Suilus Luteus *Tricholoma equestre* *Hydnum repandum*

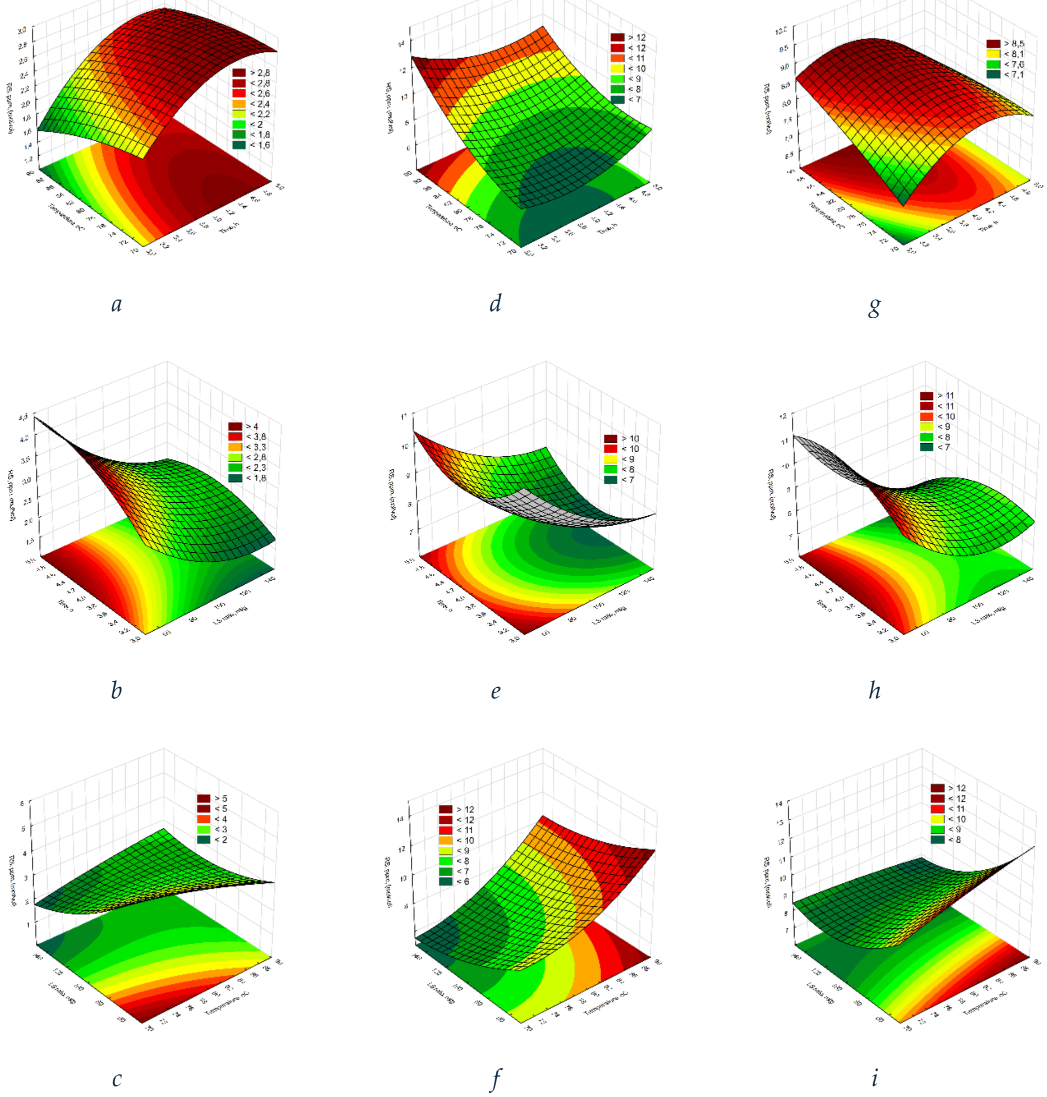

Figure 2S. Response surface plots for RS

Suilus Luteus | *Tricholoma equestre* | *Hydnum repandum*

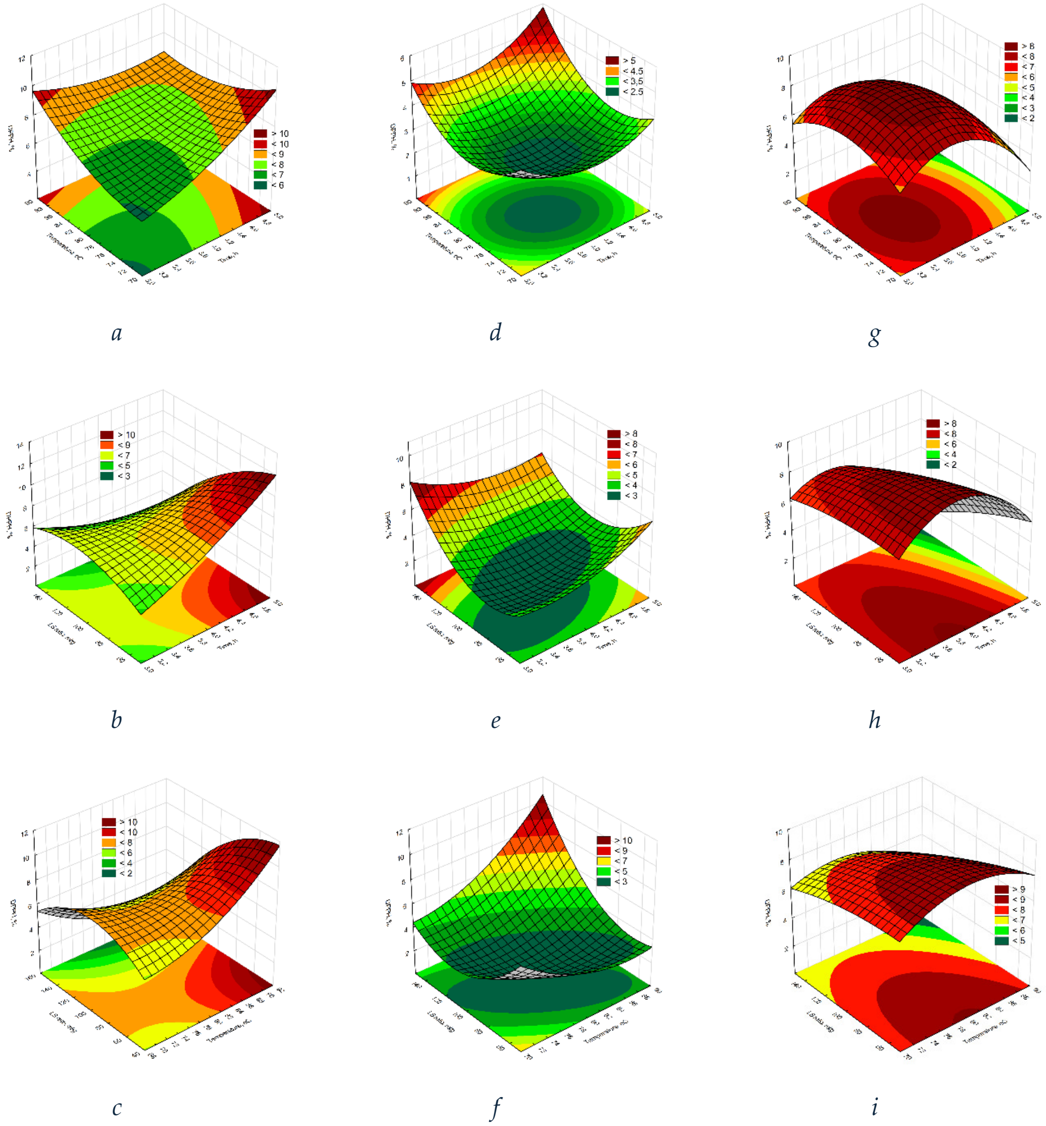

Figure 3S. Response surface plots for DPPH

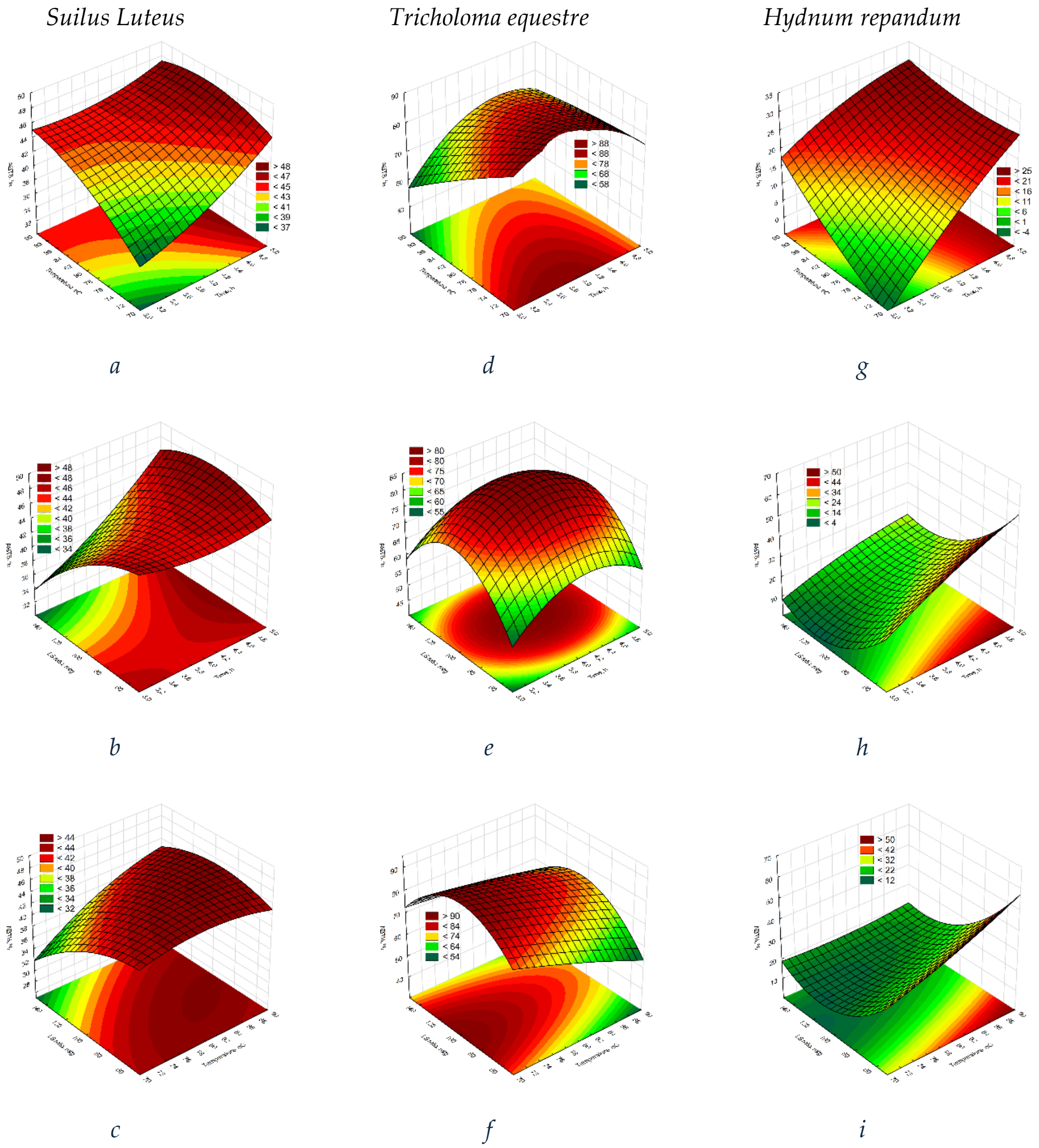

Figure 4S. Response surface plots for ABTS

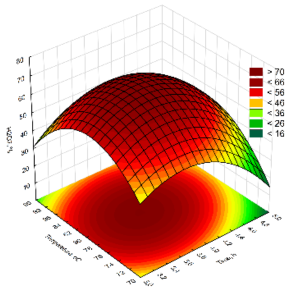

a

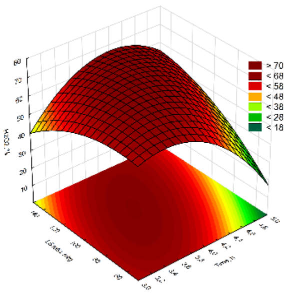

b

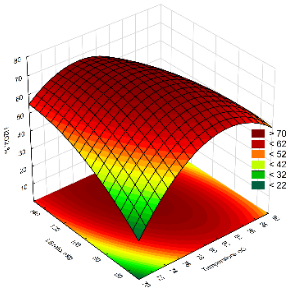

c

Figure 5S. Response surface plots for ABTS

2. Statistic data for optimization of the processes

<u>TCC model – Suillus Luteus</u>

Table S1. Model Summary

Std. Dev.	2,19
Mean	8,91
C.V. %	14,56
R^2	0,8778
Adjusted R^2	0,8629
Predicted R^2	0,8406
Adeq Precision	26,8338
Lack of Fit (p-values)	6,738E-51

Table S2. ANOVA Table

Source	Sum of Squares	df	Mean Square	F-value	p-value
Model	2547,11	9	283,01	59,06	< 0.0001
Liquid/solid Ratio	554,18	1	554,18	115,65	< 0.0001
Temperature	39,83	1	39,83	8,31	0,0052

Time	829,53	1	829,53	173,12	< 0.0001
Liquid/solid Ratio ·Temperature	16,17	1	16,17	3,37	0,0702
Time·Liquid/solid Ratio	824,09	1	824,09	171,98	< 0.0001
Time ·Temperature	16,66	1	16,66	3,48	0,0662
Liquid/solid Ratio²	162,54	1	162,54	33,92	< 0.0001
Temperature²	1,64	1	1,64	0,3427	0,5600
Time²	146,82	1	146,82	30,64	< 0.0001
Residual	354,59	74	4,79		
Pure Error	12,98	71	0,1828		
Cor Total	2901,70	83			

Table S3. Coefficients in Terms of Coded Factors

Factor	Coefficient Estimate	df	Standard Error	95% CI Low	95% CI High	VIF
Intercept	5,50	1	0,6319	4,24	6,76	
β_1	3,40	1	0,3160	2,77	4,03	1,0000
β_2	0,9109	1	0,3160	0,2814	1,54	1,0000
β_3	4,16	1	0,3160	3,53	4,79	1,0000
β_{12}	0,8208	1	0,4468	-0,0695	1,71	1,0000
β_{13}	5,86	1	0,4468	4,97	6,75	1,0000
β_{23}	0,8331	1	0,4468	-0,0573	1,72	1,0000
β_{11}	2,91	1	0,4996	1,91	3,91	1,07
β_{22}	0,2925	1	0,4996	-0,7029	1,29	1,07
β_{33}	2,77	1	0,4996	1,77	3,76	1,07

<u>TCC model – Tricholoma equestre</u>

Table S4. Model Summary

	R1
Std. Dev.	1,14
Mean	8,38
C.V. %	11,63
R^2	0,8720
Adjusted R^2	0,8391
Predicted R^2	0,7724
Adeq Precision	18,1453

Lack of Fit (p-values)	5,710E-30

Table S5. ANOVA Table

Source	Sum of Squares	df	Mean Square	F-value	p-value
Model	308,27	9	34,25	26,49	< 0.0001
Liquid/solid Ratio	0,0199	1	0,0199	0,0154	0,9020
Temperature	23,40	1	23,40	18,10	0,0001
Time	210,07	1	210,07	162,48	< 0.0001
Liquid/solid Ratio ·Temperature	0,0014	1	0,0014	0,0010	0,9743
Time·Liquid/solid Ratio	0,0013	1	0,0013	0,0010	0,9751
Time ·Temperature	43,53	1	43,53	33,67	< 0.0001
Liquid/solid Ratio^2	10,41	1	10,41	8,05	0,0075
Temperature^2	9,42	1	9,42	7,29	0,0106
Time^2	9,60	1	9,60	7,42	0,0100
Residual	45,25	74	1,29		
Pure Error	0,6117	71	0,0191		
Cor Total	353,52	83			

Table S6. Coefficients in Terms of Coded Factors

Factor	Coefficient Estimate	df	Standard Error	95% CI Low	95% CI High	VIF
Intercept	7,91	1	0,3790	7,14	8,67	
β_1	0,0288	1	0,2321	-0,4424	0,5000	1,0000
β_2	0,9874	1	0,2321	0,5162	1,46	1,0000
β_3	2,96	1	0,2321	2,49	3,43	1,0000
β_{12}	-0,0106	1	0,3282	-0,6770	0,6557	1,0000
β_{13}	0,0103	1	0,3282	-0,6560	0,6767	1,0000
β_{23}	-1,90	1	0,3282	-2,57	-1,24	1,0000
β_{11}	-0,9695	1	0,3416	-1,66	-0,2760	1,01
β_{22}	0,9223	1	0,3416	0,2287	1,62	1,01
β_{33}	0,9309	1	0,3416	0,2373	1,62	1,01

TCC model – Hydnum repandum

Table S7. Model Summary

Std. Dev.	0,2949
Mean	15,82
C.V. %	1,84
R^2	0,9976
Adjusted R^2	0,9975
Predicted R^2	0,9973
Adeq Precision	245,8889
Lack of Fit (p-values)	0,0011

Table S8. ANOVA Table

Source	Sum of Squares	df	Mean Square	F-value	p-value
Model	2856,20	3	952,07	10945,50	< 0.0001
Liquid/solid Ratio	1,99	1	1,99	22,88	< 0.0001
Temperature	1,71	1	1,71	19,65	< 0.0001
Time	2852,50	1	2852,50	32793,98	< 0.0001
Residual	6,96	74	0,0870		
Pure Error	4,79	71	0,0675		
Cor Total	2863,16	83			

Table S9. Coefficients in Terms of Coded Factors

Factor	Coefficient Estimate	df	Standard Error	95% CI Low	95% CI High	VIF
Intercept	15,82	1	0,0322	15,76	15,88	
β_1	-0,2036	1	0,0426	-0,2883	-0,1189	1,0000
β_2	-0,1887	1	0,0426	-0,2734	-0,1040	1,0000
β_3	7,71	1	0,0426	7,62	7,79	1,0000

RS model – Sillus Luteus

Table S10. Model Summary

	R1
Std. Dev.	0,3763
Mean	2,76
C.V. %	9,78
R^2	0,8764
Adjusted R^2	0,8616
Predicted R^2	0,8398
Adeq Precision	26,6774

Lack of Fit (p-values)	6,460E-15

Table S11. ANOVA Table

Source	Sum of Squares	df	Mean Square	F-value	p-value
Model	75,28	9	8,36	59,08	< 0.0001
Liquid/solid Ratio	7,60	1	7,60	53,65	< 0.0001
Temperature	2,80	1	2,80	19,75	< 0.0001
Time	44,27	1	44,27	312,72	< 0.0001
Liquid/solid Ratio ·Temperature	0,1919	1	0,1919	1,36	0,2480
Time·Liquid/solid Ratio	1,35	1	1,35	9,56	0,0028
Time ·Temperature	11,36	1	11,36	80,24	< 0.0001
Liquid/solid Ratio^2	2,67	1	2,67	18,85	< 0.0001
Temperature^2	0,1295	1	0,1295	0,9150	0,3419
Time^2	4,45	1	4,45	31,40	< 0.0001
Residual	10,62	74	0,1416		
Pure Error	4,09	71	0,0568		
Cor Total	85,90	83			

Table S12. Coefficients in Terms of Coded Factors

Factor	Coefficient Estimate	df	Standard Error	95% CI Low	95% CI High	VIF
Intercept	2,74	1	0,1086	2,53	2,96	
β_1	0,3978	1	0,0543	0,2896	0,5060	1,0000
β_2	-0,2391	1	0,0538	-0,3463	-0,1319	1,00
β_3	-0,9515	1	0,0538	-1,06	-0,8443	1,00
β_{12}	0,0894	1	0,0768	-0,0636	0,2424	1,0000
β_{13}	-0,2374	1	0,0768	-0,3904	-0,0844	1,0000
β_{23}	0,6751	1	0,0754	0,5250	0,8253	1,00
β_{11}	-0,3715	1	0,0856	-0,5419	-0,2011	1,08
β_{22}	-0,0818	1	0,0856	-0,2523	0,0886	1,07
β_{33}	0,4794	1	0,0856	0,3090	0,6498	1,07

RS model – Trichloma Equestre

Table S13. Model Summary

	R1
Std. Dev.	0,9991
Mean	8,88
C.V. %	11,14
R^2	0,7223
Adjusted R^2	0,6885
Predicted R^2	0,6426
Adeq Precision	15,6858
Lack of Fit (p-values)	0,0011

Table S14. ANOVA Table

Source	Sum of Squares	df	Mean Square	F-value	p-value
Model	192,12	9	21,35	21,38	< 0.0001
Liquid/solid Ratio	0,0958	1	0,0958	0,0959	0,7576
Temperature	4,77	1	4,77	4,78	0,0320
Time	119,94	1	119,94	120,15	< 0.0001
Liquid/solid Ratio ·Temperature	4,37	1	4,37	4,38	0,0398
Time·Liquid/solid Ratio	7,68	1	7,68	7,70	0,0070
Time ·Temperature	9,54	1	9,54	9,55	0,0028
Liquid/solid Ratio^2	20,76	1	20,76	20,79	< 0.0001
Temperature^2	2,35	1	2,35	2,35	0,1292
Time^2	14,24	1	14,24	14,26	0,0003
Residual	73,87	74	0,9982		
Pure Error	59,00	71	0,8310		
Cor Total	265,99	83			

Table S15. Coefficients in Terms of Coded Factors

Factor	Coefficient Estimate	df	Standard Error	95% CI Low	95% CI High	VIF
Intercept	9,18	1	0,2884	8,61	9,76	
β_1	-0,0447	1	0,1442	-0,3320	0,2427	1,0000
β_2	0,3152	1	0,1442	0,0278	0,6025	1,0000
β_3	-1,58	1	0,1442	-1,87	-1,29	1,0000
β_{12}	-0,4268	1	0,2039	-0,8332	-0,0205	1,0000

β_{13}	-0,5658	1	0,2039	-0,9722	-0,1594	1,0000
β_{23}	-0,6303	1	0,2039	-1,04	-0,2240	1,0000
β_{11}	-1,04	1	0,2280	-1,49	-0,5854	1,07
β_{22}	-0,3499	1	0,2280	-0,8042	0,1044	1,07
β_{33}	0,8611	1	0,2280	0,4068	1,32	1,07

<u>RS model – Hydnum Repandum</u>

Table S16. Model Summary

	R1
Std. Dev.	1,02
Mean	9,09
C.V. %	10,84
R^2	0,8400
Adjusted R^2	0,7989
Predicted R^2	0,7167
Adeq Precision	15,2816
Lack of Fit (p-values)	2,338E-21

Table S17. ANOVA Table

Source	Sum of Squares	df	Mean Square	F-value	p-value
Model	189,99	9	21,11	20,42	< 0.0001
Liquid/solid Ratio	0,4669	1	0,4669	0,4516	0,5060
Temperature	113,16	1	113,16	109,46	< 0.0001
Time	43,40	1	43,40	41,99	< 0.0001
Liquid/solid Ratio ·Temperature	6,73	1	6,73	6,51	0,0153
Time·Liquid/solid Ratio	0,1655	1	0,1655	0,1601	0,6915
Time ·Temperature	0,6622	1	0,6622	0,6405	0,4289
Liquid/solid Ratio^2	13,32	1	13,32	12,89	0,0010
Temperature^2	11,80	1	11,80	11,41	0,0018
Time^2	3,76	1	3,76	3,64	0,0646
Residual	36,18	74	1,03		
Pure Error	1,69	71	0,0528		
Cor Total	226,18	83			

Table S18. Coefficients in Terms of Coded Factors

Factor	Coefficient Estimate	df	Standard Error	95% CI Low	95% CI High	VIF
Intercept	7,64	1	0,3389	6,95	8,33	
β_1	-0,1395	1	0,2075	-0,5608	0,2819	1,0000
β_2	2,17	1	0,2075	1,75	2,59	1,0000
β_3	-1,34	1	0,2075	-1,77	-0,9235	1,0000
β_{12}	-0,7488	1	0,2935	-1,34	-0,1529	1,0000
β_{13}	0,1175	1	0,2935	-0,4784	0,7133	1,0000
β_{23}	0,2349	1	0,2935	-0,3610	0,8308	1,0000
β_{11}	1,10	1	0,3055	0,4765	1,72	1,01
β_{22}	1,03	1	0,3055	0,4119	1,65	1,01
β_{33}	0,5829	1	0,3055	-0,0373	1,20	1,01

DPPH model - Suillus Luteus

Table S19. Model Summary

	R1
Std. Dev.	1,48
Mean	7,20
C.V. %	10,05
R^2	0,8200
Adjusted R^2	0,7774
Predicted R^2	0,6948
Adeq Precision	14,2902
Lack of Fit (p-values)	2,782E-20

Table S20. ANOVA Table

Source	Sum of Squares	df	Mean Square	F-value	p-value
Model	381,45	9	42,38	19,24	< 0.0001
Liquid/solid Ratio	22,20	1	22,20	10,08	0,0030
Temperature	5,99	1	5,99	2,72	0,1074
Time	122,46	1	122,46	55,59	< 0.0001
Liquid/solid Ratio ·Temperature	24,26	1	24,26	11,01	0,0020
Time·Liquid/solid Ratio	100,41	1	100,41	45,58	< 0.0001
Time ·Temperature	33,12	1	33,12	15,03	0,0004
Liquid/solid Ratio^2	4,67	1	4,67	2,12	0,1536
Temperature^2	9,52	1	9,52	4,32	0,0444
Time^2	26,83	1	26,83	12,18	0,0012
Residual	83,71	74	2,20		

Pure Error	5,85	71	0,1671		
Cor Total	465,16	83			

Table S21. Coefficients in Terms of Coded Factors

Factor	Coefficient Estimate	df	Standard Error	95% CI Low	95% CI High	VIF
Intercept	7,11	1	0,4947	6,11	8,11	
β_1	0,9291	1	0,2927	0,3366	1,52	1,01
β_2	0,4911	1	0,2979	-0,1119	1,09	1,01
β_3	-2,15	1	0,2881	-2,73	-1,56	1,02
β_{12}	-1,42	1	0,4285	-2,29	-0,5544	1,0000
β_{13}	-2,69	1	0,3988	-3,50	-1,89	1,01
β_{23}	-1,60	1	0,4139	-2,44	-0,7670	1,01
β_{11}	0,6341	1	0,4355	-0,2476	1,52	1,03
β_{22}	0,9053	1	0,4355	0,0237	1,79	1,03
β_{33}	-1,52	1	0,4355	-2,40	-0,6383	1,02

DPPH model – Trichloma Equestre

Table S22. Model Summary

	R1
Std. Dev.	1,17
Mean	5,01
C.V. %	13,39
R^2	0,8241
Adjusted R^2	0,7789
Predicted R^2	0,6877
Adeq Precision	14,8537
Lack of Fit (p-values)	2,341E-14

Table S23. ANOVA Table

Source	Sum of Squares	df	Mean Square	F-value	p-value
Model	225,04	9	25,00	18,22	< 0.0001
Liquid/solid Ratio	0,3842	1	0,3842	0,2799	0,6001
Temperature	9,31	1	9,31	6,78	0,0134
Time	31,51	1	31,51	22,96	< 0.0001
Liquid/solid Ratio ·Temperature	1,39	1	1,39	1,01	0,3212
Time·Liquid/solid Ratio	14,05	1	14,05	10,24	0,0029

Time ·Temperature	72,81	1	72,81	53,05	< 0.0001
Liquid/solid Ratio²	11,29	1	11,29	8,23	0,0070
Temperature²	21,34	1	21,34	15,55	0,0004
Time²	73,93	1	73,93	53,86	< 0.0001
Residual	48,04	74	1,37		
Pure Error	6,16	71	0,1926		
Cor Total	273,07	83			

Table S24. Coefficients in Terms of Coded Factors

Factor	Coefficient Estimate	df	Standard Error	95% CI Low	95% CI High	VIF
Intercept	2,36	1	0,3905	1,57	3,15	
β_1	0,1265	1	0,2391	-0,3589	0,6120	1,0000
β_2	0,6227	1	0,2391	0,1372	1,11	1,0000
β_3	1,15	1	0,2391	0,6604	1,63	1,0000
β_{12}	0,3403	1	0,3382	-0,3462	1,03	1,0000
β_{13}	-1,08	1	0,3382	-1,77	-0,3955	1,0000
β_{23}	2,46	1	0,3382	1,78	3,15	1,0000
β_{11}	1,01	1	0,3520	0,2949	1,72	1,01
β_{22}	1,39	1	0,3520	0,6733	2,10	1,01
β_{33}	2,58	1	0,3520	1,87	3,30	1,01

DPPH model – Hydnum Repandum

Table S25. Model Summary

	R1
Std. Dev.	1,07
Mean	5,52
C.V. %	12,00
R^2	0,8538
Adjusted R^2	0,8210
Predicted R^2	0,7753
Adeq Precision	14,7199
Lack of Fit (p-values)	1,451E-08

Table S26. ANOVA Table

Source	Sum of Squares	df	Mean Square	F-value	p-value

Model	266,41	9	29,60	25,96	< 0.0001
Liquid/solid Ratio	78,04	1	78,04	68,45	< 0.0001
Temperature	5,16	1	5,16	4,53	0,0396
Time	45,25	1	45,25	39,69	< 0.0001
Liquid/solid Ratio ·Temperature	0,8380	1	0,8380	0,7351	0,3963
Time·Liquid/solid Ratio	9,99	1	9,99	8,76	0,0052
Time ·Temperature	4,61	1	4,61	4,04	0,0511
Liquid/solid Ratio^2	90,97	1	90,97	79,80	< 0.0001
Temperature^2	25,40	1	25,40	22,28	< 0.0001
Time^2	1,64	1	1,64	1,44	0,2378
Residual	45,60	74	1,14		
Pure Error	15,94	71	0,4308		
Cor Total	312,01	83			

Table S27. Coefficients in Terms of Coded Factors

Factor	Coefficient Estimate	df	Standard Error	95% CI Low	95% CI High	VIF
Intercept	8,21	1	0,4036	7,39	9,02	
β_1	-1,68	1	0,2033	-2,09	-1,27	1,02
β_2	-0,4270	1	0,2007	-0,8325	-0,0214	1,02
β_3	-1,26	1	0,2002	-1,67	-0,8565	1,02
β_{12}	-0,2459	1	0,2868	-0,8256	0,3338	1,01
β_{13}	-0,8534	1	0,2883	-1,44	-0,2707	1,01
β_{23}	-0,5666	1	0,2818	-1,14	0,0029	1,03
β_{11}	-2,85	1	0,3190	-3,49	-2,20	1,10
β_{22}	-1,51	1	0,3195	-2,15	-0,8623	1,09
β_{33}	-0,3823	1	0,3190	-1,03	0,2624	1,09

ABTS model – Suillus Luteus

Table S28. Model Summary

	R1
Std. Dev.	3,16
Mean	42,61
C.V. %	6,85
R^2	0,7178

Adjusted R^2	0,6573
Predicted R^2	0,5558
Adeq Precision	11,2287
Lack of Fit (p-values)	0,0229

Table S29. ANOVA Table

Source	Sum of Squares	df	Mean Square	F-value	p-value
Model	1065,93	9	118,44	11,87	< 0.0001
Liquid/solid Ratio	249,19	1	249,19	24,97	< 0.0001
Temperature	198,05	1	198,05	19,85	< 0.0001
Time	233,87	1	233,87	23,44	< 0.0001
Liquid/solid Ratio ·Temperature	45,65	1	45,65	4,58	0,0383
Time·Liquid/solid Ratio	156,65	1	156,65	15,70	0,0003
Time ·Temperature	101,14	1	101,14	10,14	0,0027
Liquid/solid Ratio^2	15,08	1	15,08	1,51	0,2257
Temperature^2	32,61	1	32,61	3,27	0,0778
Time^2	65,66	1	65,66	6,58	0,0140
Residual	419,07	74	9,98		
Pure Error	329,14	71	8,44		
Cor Total	1485,00	83			

Table S30. Coefficients in Terms of Coded Factors

Factor	Coefficient Estimate	df	Standard Error	95% CI Low	95% CI High	VIF
Intercept	44,02	1	1,12	41,76	46,27	
β_1	2,91	1	0,5824	1,74	4,09	1,02
β_2	2,65	1	0,5944	1,45	3,85	1,03
β_3	-2,89	1	0,5965	-4,09	-1,68	1,03
β_{12}	-1,75	1	0,8196	-3,41	-0,0991	1,01
β_{13}	3,30	1	0,8336	1,62	4,99	1,03
β_{23}	2,74	1	0,8591	1,00	4,47	1,03
β_{11}	1,13	1	0,9162	-0,7225	2,98	1,07
β_{22}	-1,65	1	0,9136	-3,50	0,1920	1,07
β_{33}	-2,35	1	0,9165	-4,20	-0,5015	1,08

ABTS model – Trichloma Equestre

Table S31. Model Summary

	R1
Std. Dev.	5,53
Mean	69,09
C.V. %	7,98
R^2	0,8446
Adjusted R^2	0,8035
Predicted R^2	0,7238
Adeq Precision	12,2430
Lack of Fit (p-values)	1,319E-18

Table S32. ANOVA Table

Source	Sum of Squares	df	Mean Square	F-value	p-value
Model	5659,49	9	628,83	20,54	< 0.0001
Liquid/solid Ratio	70,77	1	70,77	2,31	0,1377
Temperature	1522,64	1	1522,64	49,73	< 0.0001
Time	48,72	1	48,72	1,59	0,2158
Liquid/solid Ratio ·Temperature	497,59	1	497,59	16,25	0,0003
Time·Liquid/solid Ratio	4,78	1	4,78	0,1562	0,6951
Time ·Temperature	176,42	1	176,42	5,76	0,0220
Liquid/solid $Ratio^2$	949,88	1	949,88	31,02	< 0.0001
$Temperature^2$	7,20	1	7,20	0,2351	0,6309
$Time^2$	2670,08	1	2670,08	87,20	< 0.0001
Residual	1041,03	74	30,62		
Pure Error	66,43	71	2,14		
Cor Total	6700,52	83			

Table S33. Coefficients in Terms of Coded Factors

Factor	Coefficient Estimate	df	Standard Error	95% CI Low	95% CI High	VIF
Intercept	83,31	1	1,96	79,34	87,29	
β_1	1,72	1	1,13	-0,5782	4,01	1,0000
β_2	-7,97	1	1,13	-10,26	-5,67	1,0000
β_3	1,42	1	1,13	-0,8707	3,72	1,0000
β_{12}	6,44	1	1,60	3,19	9,69	1,0000
β_{13}	-0,6313	1	1,60	-3,88	2,61	1,0000
β_{23}	3,83	1	1,60	0,5881	7,08	1,0000
β_{11}	-9,44	1	1,69	-12,88	-5,99	1,02
β_{22}	-0,8215	1	1,69	-4,26	2,62	1,02

β_{33}	-15,82	1	1,69	-19,26	-12,38	1,02

ABTS model – Hydnum Repandum

Table S34. Model Summary

	R1
Std. Dev.	8,25
Mean	25,14
C.V. %	13,14
R^2	0,8209
Adjusted R^2	0,7851
Predicted R^2	0,7234
Adeq Precision	18,0039
Lack of Fit (p-values)	8,079E-27

Table S35. ANOVA Table

Source	Sum of Squares	df	Mean Square	F-value	p-value
Model	14050,45	9	1561,16	22,92	< 0.0001
Liquid/solid Ratio	3716,62	1	3716,62	54,56	< 0.0001
Temperature	1400,10	1	1400,10	20,55	< 0.0001
Time	4925,56	1	4925,56	72,31	< 0.0001
Liquid/solid Ratio ·Temperature	380,08	1	380,08	5,58	0,0226
Time·Liquid/solid Ratio	180,92	1	180,92	2,66	0,1101
Time ·Temperature	565,96	1	565,96	8,31	0,0060
Liquid/solid Ratio^2	53,32	1	53,32	0,7828	0,3810
Temperature^2	33,56	1	33,56	0,4927	0,4864
Time^2	2158,52	1	2158,52	31,69	< 0.0001
Residual	3065,28	74	68,12		
Pure Error	162,24	71	3,86		
Cor Total	17115,73	83			

Table S36. Coefficients in Terms of Coded Factors

Factor	Coefficient Estimate	df	Standard Error	95% CI Low	95% CI High	VIF
Intercept	17,34	1	2,92	11,46	23,21	
β_1	11,41	1	1,55	8,30	14,53	1,01

β_2	6,61	1	1,46	3,67	9,54	1,03
β_3	-12,61	1	1,48	-15,60	-9,62	1,03
β_{12}	-5,06	1	2,14	-9,37	-0,7453	1,01
β_{13}	-3,61	1	2,22	-8,07	0,8518	1,01
β_{23}	-5,78	1	2,00	-9,81	-1,74	1,04
β_{11}	-2,08	1	2,35	-6,82	2,66	1,11
β_{22}	1,64	1	2,34	-3,07	6,36	1,06
β_{33}	13,21	1	2,35	8,48	17,93	1,08

H_2O_2 model – Hydnum Repandum

Table S37. Model Summary

	R1
Std. Dev.	8,22
Mean	43,33
C.V. %	9,98
R^2	0,8540
Adjusted R^2	0,8203
Predicted R^2	0,7624
Adeq Precision	14,7747
Lack of Fit (p-values)	1,900E-14

Table S38. ANOVA Table

Source	Sum of Squares	df	Mean Square	F-value	p-value
Model	15425,31	9	1713,92	25,34	< 0.0001
Liquid/solid Ratio	1400,06	1	1400,06	20,70	< 0.0001
Temperature	20,11	1	20,11	0,2973	0,5887
Time	464,79	1	464,79	6,87	0,0124
Liquid/solid Ratio ·Temperature	426,93	1	426,93	6,31	0,0162
Time·Liquid/solid Ratio	3193,60	1	3193,60	47,21	< 0.0001
Time ·Temperature	2177,67	1	2177,67	32,19	< 0.0001
Liquid/solid Ratio^2	3851,43	1	3851,43	56,94	< 0.0001
Temperature^2	5926,26	1	5926,26	87,61	< 0.0001

$Time^2$	688,46	1	688,46	10,18	0,0028
Residual	2638,02	39	67,64		
Pure Error	419,46	36	11,65		
Cor Total	18063,33	48			

Table S39. Coefficients in Terms of Coded Factors

Factor	Coefficient Estimate	df	Standard Error	95% CI Low	95% CI High	VIF
Intercept	69,42	1	2,91	63,54	75,30	
β_1	-7,26	1	1,60	-10,49	-4,03	1,02
β_2	0,8681	1	1,59	-2,35	4,09	1,01
β_3	4,11	1	1,57	0,9378	7,27	1,01
β_{12}	5,76	1	2,29	1,12	10,40	1,01
β_{13}	15,18	1	2,21	10,71	19,65	1,01
β_{23}	-12,60	1	2,22	-17,09	-8,11	1,01
β_{11}	-18,30	1	2,43	-23,20	-13,39	1,05
β_{22}	-22,70	1	2,43	-27,61	-17,80	1,05
β_{33}	-7,74	1	2,43	-12,64	-2,83	1,04
